# Neural correlates of video game empathy training in adolescents: a randomized trial

**DOI:** 10.1038/s41539-018-0029-6

**Published:** 2018-08-07

**Authors:** Tammi R. A. Kral, Diane E. Stodola, Rasmus M. Birn, Jeanette A. Mumford, Enrique Solis, Lisa Flook, Elena G. Patsenko, Craig G. Anderson, Constance Steinkuehler, Richard J. Davidson

**Affiliations:** 1 Center for Healthy Minds, University of Wisconsin, Madison, USA; 2 Department of Psychology, University of Wisconsin, Madison, USA; 3 Waisman Laboratory for Brain Imaging and Behavior, University of Wisconsin, Madison, USA; 4 Department of Psychiatry, University of Wisconsin, Madison, USA; 5 Department of Medical Physics, University of Wisconsin, Madison, USA; 6 Games+Learning+Society, University of Wisconsin, Madison, USA; 7 Department of Curriculum and Instruction, University of Wisconsin, Madison, USA; 8 Department of Informatics, University of California, Irvine, USA

## Abstract

The ability to understand emotional experiences of others, empathy, is a valuable skill for effective social interactions. Various types of training increase empathy in adolescents, but their impact on brain circuits underlying empathy has not been examined. Video games provide a unique medium familiar and engaging to adolescents and can be used to deliver training at scale. We developed an empathy training video game, Crystals of Kaydor (Crystals), and investigated whether playing Crystals increases empathic accuracy (EA) and related brain activation in adolescents (*N* = 74; 27 female; mean age(sd) = 12.8(0.7) years; age range 11–14 years). Participants completed a resting state functional MRI (rs-fMRI) scan and an EA task during an fMRI scan before and after 2 weeks of daily gameplay with either the empathy training game, Crystals (*N* = 34), or the commercial video game Bastion (*N* = 40), an active control condition. There were no group differences in EA improvement following gameplay, however, engagement with training aspects of Crystals was associated with a higher increase in EA-related activation in right temporoparietal junction following gameplay. Moreover, rs-fMRI connectivity in empathy-related brain circuits (posterior cingulate–medial prefrontal cortex; MPFC) was stronger after Crystals gameplay compared to Bastion. The more individuals’ EA increased following Crystals versus Bastion, the stronger their rs-fMRI connectivity in brain circuits relevant for emotion regulation (amygdala-MPFC). These findings suggest that a video game designed to increase empathic accuracy produces behaviorally-relevant, functional neural changes in fewer than 6 h of gameplay in adolescents.

## Neuroscience: Video game strengthens empathy-related brain networks

A video game designed to train empathy in middle schoolers can produce behaviorally-relevant changes in the brain in as little as two weeks. Tammi Kral, Richard Davidson and researchers at the University of Wisconsin–Madison gauged volunteers’ empathy by conducting brain scans and assessing volunteers’ ratings of others’ emotions before and after playing a video game that teaches empathy (Crystals of Kaydor) or a control game (Bastion). Scientists discovered that a video game (in this case, Crystals of Kaydor) could strengthen empathy-related brain circuits. Video games are a popular activity for youth, and this research suggests that specially-designed games may be an engaging and effective medium for improving empathy-related brain function during adolescence – a time of increased brain development and susceptibility to mental health disorders.

## Introduction

Adolescence is a developmental period marked by profound physical and psychological change. Many individuals experience their first onset of psychiatric illness such as anxiety and depression during this time.^1,2^ Further exacerbating what can be a difficult transition, a recent meta-analysis found that approximately 36% of adolescents were victims of bullying,^3^ which is associated with psychosocial adjustment problems,^4^ greater suicidal ideation and suicide attempts,^5^ and serious health consequences, such as increased systemic inflammation that can persist into adulthood.^6^ Research indicates that empathy is a skill that can buoy well-being by reducing the negative impact of poor peer relations on personal adjustment—young adolescents with higher empathic accuracy (a behavioral measure of empathic ability) have less internalizing symptoms and are less likely to be targets of bullying.^7^ Furthermore, the ability to form positive, supportive relationships can buffer the development of both psychological and physical suffering, particularly in the presence of stress.^8^ Thus, training that increases empathy may be particularly beneficial during adolescence.

In order to effectively assess the impact of training programs for improving empathic abilities it is critical to utilize measures as similar as possible to real-world situations. The empathic accuracy task is one of the more ecologically valid measures of empathy, as the stimuli consist of videos of people (“targets”) discussing emotional life events. This task requires identifying the target’s emotions in line with the target’s ratings of their own emotions. Participants are instructed to rate how the target is feeling in the video rather than to use a specific strategy, and are thus able to utilize either or both of the processes found to underlie empathy: perspective taking and experience sharing.^9^ The empathic accuracy task is unique in that the videos depict the target’s changing emotional expressions, which provides an opportunity for mimicry, and also a narrative component that allows participants to understand the event from the target’s perspective. Conversely, much of the literature on empathy is based on research that examines the cognitive and affective aspects of empathy separately (i.e., perspective taking and experience sharing, respectively), for example using cartoon vignettes or images of bodily injury.^10,11^

Separable neural networks underlie perspective taking and experience sharing,^9,12–15^ and these networks are both activated when adults are empathically accurate.^16^ However, as we previously reported, adolescents only activated regions in the network underlying perspective taking when making empathically accurate responses. These regions included right temporoparietal junction (RTPJ), ventral and dorsal medial prefrontal cortex (VMPFC and DMPFC, respectively). The RTPJ and DMPFC participate in a sub-network of the brain’s default mode network during the resting state,^17^ which has also been implicated in taking a third person perspective more generally.^10,18–21^ In contrast, activation in regions related to experience sharing, including right anterior insula and right inferior parietal lobule, related to less empathic accuracy in adolescents.^22^ One interpretation of this negative brain-behavior relationship is that the adolescents’ accuracy was worse due to their experience of empathic distress when sharing the target’s emotions, as experience sharing can lead to empathic distress, particularly when emotion regulation is lacking.^23–25^ Notably, a large body of research indicates that adolescents have difficulty with emotion regulation.^26^ This may indicate that adolescents are better able to empathize when utilizing perspective taking than experience sharing, possibly due to an inability to regulate the emotions associated with sharing another’s emotional experience. One way to address this potential developmental shortcoming is through training to increase the use of perspective taking as a strategy, and to improve implicit emotion regulation.

The potential to train empathic accuracy abilities in adolescents finds support in research by Goldstein and Winner.^27^ They found that students in an intense acting class that utilized role-playing had increased performance on an empathic accuracy task compared to an active control group. Students in the control group received equally intensive training in either visual arts or music. While an important step in providing evidence for the feasibility of training empathy in adolescence, this study required participants to attend a special arts school. It is important to develop and test additional modes by which to deliver training.

An intervention could have a broad impact by being accessible, convenient, and engaging, in addition to utilizing effective training. Delivering training with an interactive, immersive video game on a mobile device is one way to accomplish this goal. Adolescents consume video games daily, as a survey by the Kaiser Family Foundation found that American children between the ages of 8 and 18 years old spend an average of 73 min per day playing video games (2010).^28^ Work by Greitemeyer and Osswald provides additional support for the efficacy of video games as an educational platform.^29^ They demonstrated that playing a commercially available video game (Lemmings) with a pro-social context increased pro-social behavior. They proposed a mechanism whereby video games encourage learning by providing environments with opportunities for modeling, rehearsal and reinforcement.^29^ Therefore, pro-social video games may provide a unique opportunity to deliver training in a format that adolescents are already utilizing, and that can be both engaging and effective. However, the Lemmings video game was created for entertainment purposes, and while it includes a pro-social context, the game mechanics solely involve problem solving needed to pass through a set of mazes. In the current study we sought to create a game with mechanics that directly address processes underlying empathy.

**Fig. 1:**
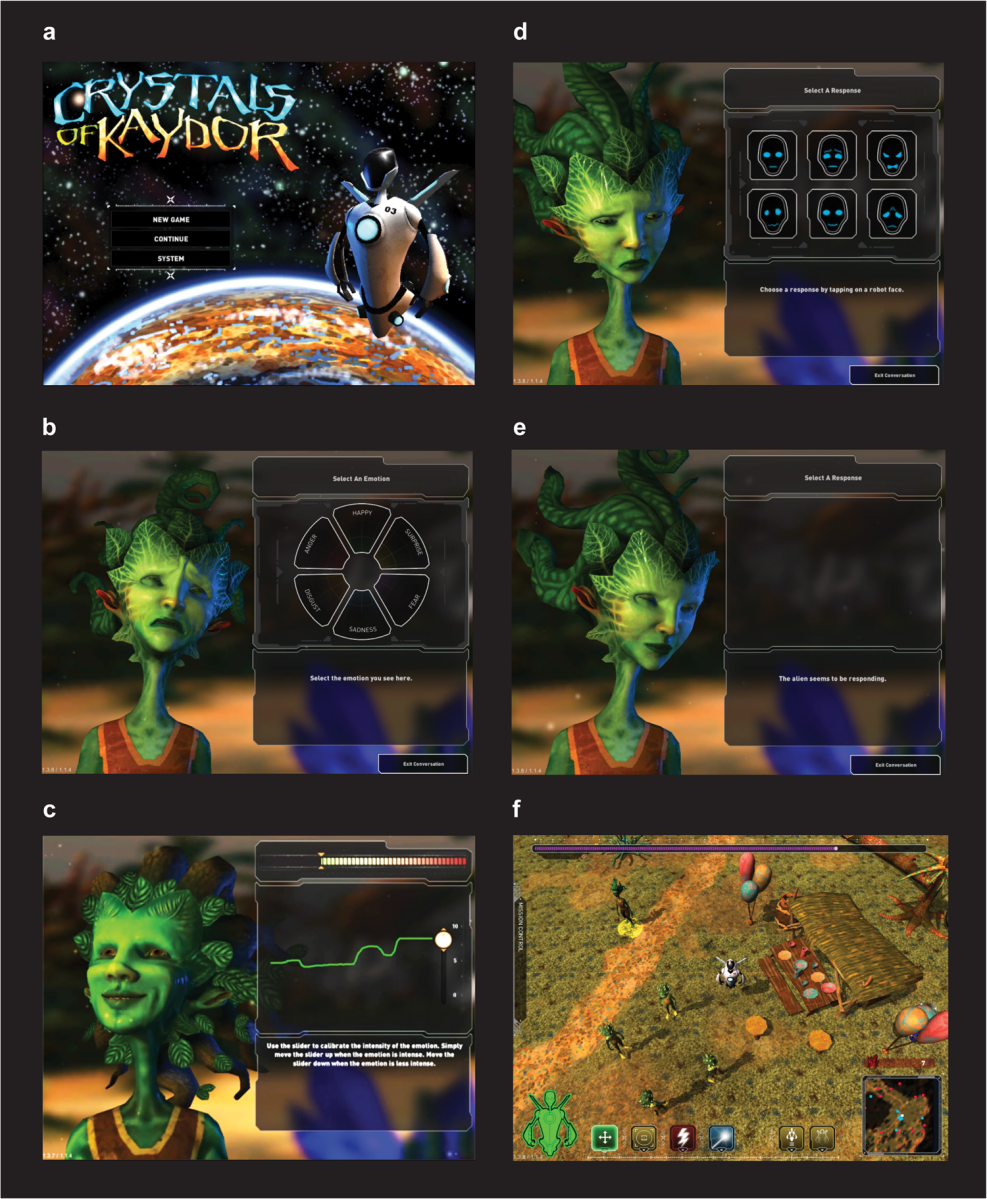
Crytsals of Kaydor empathy training game. Title screen depicting the character that a player controls from the third person perspective (**a**), emotion recognition game mechanic where the player selects which of the 6 basic emotions (happy, surprise, sadness, disgust, fear or anger) is shown by the animated alien avatar (**b**), emotion intensity calibration with a slider that the player moves up and down to gauge the changing intensity of the avatar’s emotion (**c**), empathic responding mechanic where the player must select one of 6 iconic emotional expressions to communicate their empathy to the avatar (**d**), the interactive response of the avatar where the animated emotional expression changes following a player’s empathic response in the previous panel (**e**), and the helping quest in which the player engages in a mission such as helping the avatars find missing items (**f**)

In order to test whether an empathy video game intervention would improve empathic accuracy and underlying brain activation and network connectivity, we developed a videogame with empathy training mechanics: Crystals of Kaydor (Crystals, Fig. 1; for a video overview see https://gearlearning.org/microsites/kaydor/). Crystals is a story-based iPad game to train empathy with cutting-edge animation based on the Facial Action Coding System.^30^ Players learn to recognize six basic emotions (anger, fear, happiness, surprise, disgust and sadness) by paying attention to the avatars’ facial expressions and head movements (Fig. 1b). Crystals also includes an in-game training tool in which players gauge the intensity of emotions by moving a slider up for strong intensity and down for low intensity, thereby learning how to identify emotions across a continuum from subtle to strong (Fig. 1c). Players receive feedback on their accuracy and can re-play the empathy game mechanics, providing additional opportunities for training and rehearsal that has been proposed as a mechanism for learning and transfer of pro-social skills from video games to real life.^29^ When players have successfully mastered the ability to identify and gauge the six basic emotions they have the opportunity to respond emotionally to the avatar by choosing one of six iconic facial expressions (Fig. 1d). Following an empathically appropriate response the player receives interactive feedback through a change in the emotional expression of the avatar (Fig. 1e), which provides positive reinforcement hypothesized to aid in video game-based learning.^29^ The positive reinforcement is two-fold, as players receive the positive social experience of the avatar smiling, as well as the rewarding experience of winning by solving the problem correctly. Positive social reinforcement has been shown to share behavioral and neural properties with basic reinforcement reward learning modeled in non-human primates.^31^ As players progress through the game they encounter opportunities to go on quests in which they help the avatars that reside in the game world, and in doing so learn how the narrative context impacts the avatars’ emotions. The emotional narratives provide cues and implicit motivation to take the avatars’ perspectives to improve performance on the empathy training aspects of the game. If players engage with the quests as expected and use the narratives to inform their responses on emotion recognition and intensity ratings this rehearsal may activate and strengthen perspective taking behavior and the underlying brain networks. The quests include activities such as finding missing birthday presents (Fig. 1f) and sharing a ball with an excluded child avatar. These quests drive the narrative of the game, allowing the player to earn the avatars’ trust and progress to new areas, while simultaneously encouraging the player to practice emotion recognition, emotion intensity ratings and empathic responding.

In the current study we sought to test whether a video game designed to train empathy (Crystals) would improve empathic accuracy, empathy-related brain activation and connectivity of brain networks relevant for empathic processing in adolescents. Game-play data that tracked participants performance and in-game behavior was used to address the question of how engagement with different game mechanics impacted changes in empathic behavior, brain activation and connectivity. We hypothesized that participants who played the empathy training game Crystals would show greater empathic accuracy on a behavioral task, and higher self-reported empathic concern following training (as measured by the Interpersonal Reactivity Index, IRI)^32^ compared to participants in a control group who played a similar style commercial game, Bastion, which did not include specific mechanics for training empathy. We predicted that increased empathic accuracy would be associated with greater empathic concern. It is sometimes the case that while group differences in the main effect of interest are not present, those participants in the experimental intervention group who showed the hypothesized neural change would be expected to show the relevant behavioral change. Accordingly, we predicted that increases in empathic accuracy should be accompanied by, and related to, increased activation during the empathic accuracy task and increased resting connectivity for the neural regions underlying empathic accuracy in adolescents, including VMPFC, DMPFC and RTPJ.^22^ We interrogated individual differences in gameplay for participants who played Crystals and hypothesized that participants with greater engagement with and performance on the empathy training game mechanics (as measured by telemetry data collected from the game) would show the most change in empathic accuracy and related brain activation. Since we previously found that participants had higher activation in a network of regions related to perspective taking when they had higher empathic accuarcy (at baseline), and since Crystals may have encouraged players to practice perspective taking (as described above) we expected that training adolescents to more accurately empathize would be supported and reflected by strengthened connectivity wihin this network. We tested for changes in the strength of rs-fMRI connectivity between seed regions related to empathic accuracy in our prior work (VMPFC, DMPFC and RTPJ) with the rest of the empathic accuracy-related network based on an independent sample (participants who were randomized to a different pair of video games not under investigation in this study), as well as with networks relevant for emotion regulation (VMPFC—amygdala and DMPFC—amygdala) since our prior work indicated a potential interaction between emotion regulation ability and empathic accuracy as an area where adolescents may also benefit from improvement.^22^ We also examined whether changes in connectivity had behaviorally relevant consequences in the form of changes in empathic accuracy.

## Results

All results are reported after removing outliers based on Cook’s D using a cutoff threshold of 4/(*N*−*P*) for data points disconnected from the distribution (where *N* = sample size and *P* = number of parameters in the model). When outliers were removed from the model they were also removed from the corresponding figure and table. In order to determine whether the empathy training game Crystals improved empathic accuracy and related brain activation, we compared the change from pre-training to post-training measures (time 2 − time 1 difference scores) in participants randomized to play Crystals with the active control condition (the commercially available video game Bastion). We also conducted follow-up tests for each group comparison to determine whether there were significant effects within either group, and confirmed that there were no baseline differences between groups on any of the dependent variables. Significant results from the region of interest (ROI) analyses all survive family-wise correction for multiple comparisons using the Holm-Bonferroni method across the set of ROIs used for each test.^33^ Voxel-wise analyses were thresholded at a family-wise error of *p* < 0.05 using threshold-free cluster enhancement with FSL’s Randomize.^34^ Descriptive statistics for variables described in the following results sections are provided in Table 1.

**Table 1:** Descriptive statistics

Measure	Group	*N*	Mean	S.D.	Min.	Max.
EA delta	Crystals	33	−0.03	0.21	−0.39	0.40
	Bastion	40	0.03	0.25	−0.43	0.47
EC delta	Crystals	31	0.06	2.57	−6	5
	Bastion	39	0.03	2.49	−5	6
VMPFC – Amygdala rs-fMRI delta	Crystals	31	0.10	0.43	−0.75	0.88
	Bastion	33	−0.03	0.28	−0.76	0.54
DMPFC – Amygdala rs-fMRI delta	Crystals	30	0.10	0.43	−0.83	1.20
	Bastion	33	0.04	0.35	−0.79	0.83
RTPJ EA-related activation delta	Crystals	33	−0.02	0.66	−1.75	1.69
	Bastion	39	−0.05	1.13	−3.55	1.83
VMPFC EA-related activation delta	Crystals	33	−0.03	1.08	−3.04	3.15
	Bastion	38	−0.26	1.43	−5.27	2.67
DMPFC EA-related activation delta	Crystals	33	−0.29	0.56	−2.09	0.85
	Bastion	39	0.05	1.00	−3.14	3.15
Emotion identification first tries	Crystals	33	54.0	12.0	27	74
Emotion intensity first tries	Crystals	33	29.6	10.4	14	60
Game engagement	Crystals	33	0.06	0.92	−1.72	2.24
Total time playing Crystals	Crystals	33	220.2	53.2	121.0	334.1
Number taser uses	Crystals	33	19.3	18.0	3	103
Number quests completed	Crystals	33	36.1	6.7	19	57

Descriptive statistics by group for all variables used in analysis. *S.D.* standard deviation, *Min.* minimum, *Max.* maximum, delta, time 2 – time 1 difference, *EA* empathic accuracy, *EC* self-reported empathic concern, *rs-fMRI* resting state functional magnetic resonance imaging, *VMPFC* ventromedial prefrontal cortex, *DMPFC* dorsomedial prefrontal cortex, *RTPJ* right temporoparietal junction

### Group differences in behavior and self-report

Group differences in the post- minus pre-intervention empathic accuracy scores were tested using a two-sample t-test to examine the effect of video game training on behavior. There was no significant group difference in empathic accuracy change (*t*(71) = −1.04, *p* = 0.30, *b* = −0.06, CI = [−0.16, 0.05]; 1 Crystals outlier removed; Supplementary Figure 1). Nor was there a change in empathic accuracy from pre-intervention to post-intervention within Crystals (*t*(32) = -0.78, *p* = 0.44, *b* = -0.03, CI = [-0.10, 0.05]; 1 Crystals outlier removed) or Bastion participants (*t*(39) = 0.71, *p* = 0.48, *b* = 0.03, CI = [−0.05, 0.11]).

We also tested for relationships between self-reported empathic concern and our behavioral measure of empathic accuracy. First, we confirmed that there was a relationship between empathic accuracy and self-reported empathic concern across all participants at baseline, such that participants with higher empathic accuracy had higher self-reported empathic concern (*t*(72) = 2.47, *p* = 0.016, *b* = 4.64, CI = [0.90, 8.39], *r* = 0.28). We tested whether there was an association between changes in empathic accuracy and empathic concern, and found support for this hypothesis (*t*(70) = 2.23, *p* = 0.03, *b* = 2.99, CI = [0.31, 5.67], *r* = 0.30; 1 Bastion outlier removed; Supplementary Figure 2). There was no group difference in change in self-reported empathic concern (*t*(68) = 0.15, *p* = 0.88, *b* = 0.09, CI = [−1.12, 1.31]; 1 Bastion and 2 Crystals outliers removed).

### Training-related change in rs-fMRI connectivity: voxel-wise analyses

**Fig. 2:**
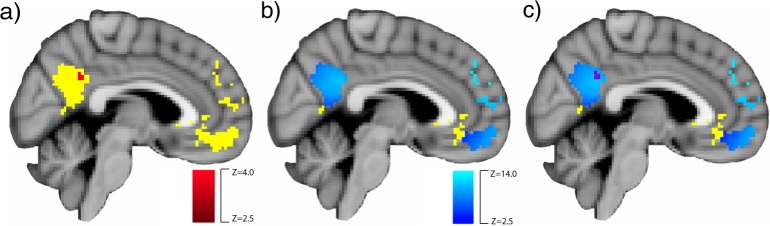
Training-related increases in DMPFC—PCC rs-fMRI connectivity after Crystals relative to Bastion. The significant group difference in change in DMPFC—PCC rs-fMRI connectivity is in red (**a**), the significant difference (time 2 − time 1) within Crystals participants is in blue (**b**), and the overlap between the two contrasts is depicted in purple (**c**). Results are overlaid on the independently-defined functional mask in yellow in Montreal Neurological Institute template space at slice *x* = 43, thresholded and corrected for family-wise error at *p* < 0.05

We examined whether playing Crystals led to increased connectivity between each of the three seed ROIs used in the task-based analysis (VMPFC, DMPFC, and RPTJ) within the network of regions where activation during the EA task related to higher empathic accuracy (and which have been related to perspective taking).^15,16,20,22,35^ We defined the target mask based on the same independent sample of participants whose brain activation was used to define the seed ROIs, and conducted a voxel-wise analysis within the mask excluding the relevant seed regions within each analysis. Participants who played Crystals had increased DMPFC – posterior cingulate rs-fMRI connectivity following game-play, relative to participants who played Bastion (Fig. 2a, in red). Within the Crystals group, there was increased rs-fMRI connectivity between this network of regions (Fig. 2b in blue), and there were no regions where Bastion participants had significantly stronger DMPFC rs-fMRI connectivity following game-play than the Crystals group. Changes in DMPFC connectivity within the empathic accuracy-related network mask were not related to changes in empathic accuracy for Crystals participants (*t*(28) = 1.22, *p* = 0.23, *b* = 0.15, CI = [−0.10, 0.40]; 1 outlier removed). There were no significant group differences in changes for VMPFC or RTPJ rs-fMRI connectivity following game-play.

We also tested for changes in rs-fMRI connectivity between the 3 seed ROIs (VMPFC, DMPFC and RTPJ) in a whole-brain, voxel-wise analysis to determine whether there were other regions where connectivity changed following gameplay. There were no significant group differences in change in rs-fMRI connectivity in the whole-brain analysis. Since rs-fMRI data is particularly susceptible to motion artifacts,^36^ we also verified that there were no group differences in average motion across scans (*t*(67) = 0.33, *p* = 0.74, *b* *=* 1.92, CI = [−9.8, 13.6]), or in the difference in motion from pre-intervention to post-intervention (*t*(67) = 0.38, *p* = 0.70, *b* *=* 4.31, CI = [−18.2, 26.9]).

### Empathic accuracy in relation to rs-fMRI connectivity: ROI analyses

**Fig. 3:**
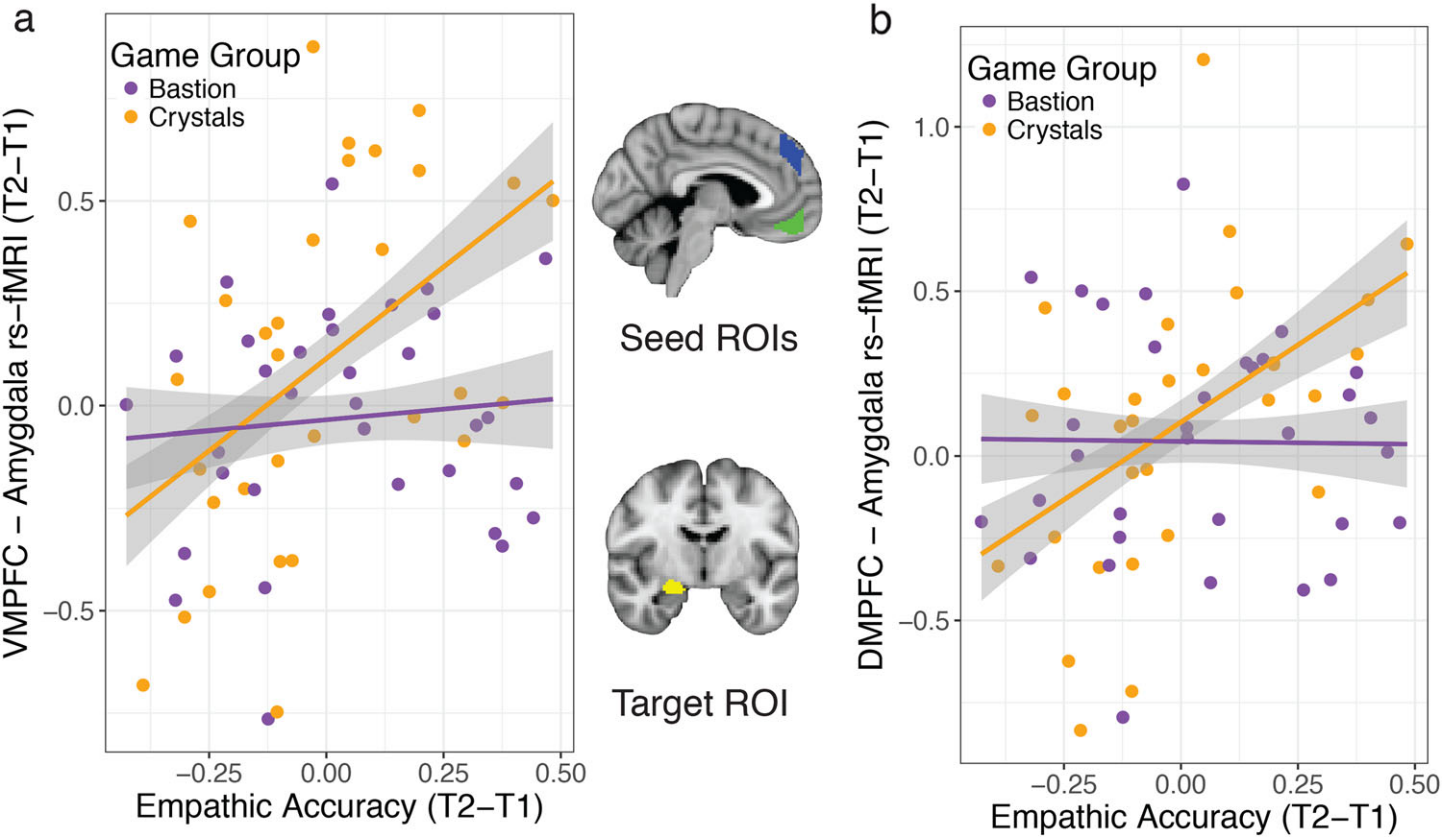
Game-related changes in empathic accuracy were associated with rs-fMRI connectivity changes in emotion regulation networks. Participants who improved in empathic accuracy had increased right amygdala rs-fMRI connectivity with VMPFC (**a**) and DMPFC (**b**) after Crystals game-play compared to Bastion. Independently-defined functional seed ROIs are overlaid in blue (DMPFC) and green (VMPFC) at slice *x* = 47 in Montreal Neurological Institute (MNI) template space. The Harvard-Oxford atlas right amygdala target ROI is depicted in yellow at slice *y* = 59 in MNI space. Envelopes represent 1 standard error from the point estimates and raw data points are overlaid

Given the potential interaction between emotion regulation ability and empathic accuracy, we tested whether changes in rs-fMRI connectivity of brain networks related to emotion regulation (VMPFC or DMPFC with right amygdala) were associated with improvements in empathic accuracy following Crystals game-play compared to Bastion. Improved empathic accuracy was associated with increased right amygdala–VMPFC rs-fMRI connectivity (T2−T1) in participants who played Crystals (t(29) = 2.95, *p* = 0.01, *b* = 0.90, CI = [0.27, 1.52]). The relationship between changes in empathic accuracy and VMPFC – right amygdala rs-fMRI was significantly greater for Crystals versus Bastion (*t*(60) = 2.23, *p* = 0.03, *b* = 0.79, CI = [0.08, 1.50], 1 Bastion outlier removed; Fig. 3a), as there was no statistically significant relationship in the Bastion group (*t*(31) = 0.10, *p* = 0.60, *b* = 0.10, CI = [−0.30, 0.51]). There was no group difference in the change in VMFPC—right amygdala rs-fMRI connectivity (*t*(59) = 1.46, *p* = 0.15, *b* = 0.12, CI = [−0.04, 0.28], 1 Bastion and 3 Crystals outliers removed). There was no difference in VMPFC – right amygdala rs-fMRI connectivity between groups at baseline (*t*(61) = -0.55, *p* = 0.59, *b* = −0.04, CI = [−0.17, 0.10], 2 Crystals outliers removed), nor in the relationship between empathic accuracy and VMPFC—right amygdala rs-fMRI at baseline (*t*(60) = −0.85, *p* = 0.40, *b* = −0.36, CI = [-1.22, 0.49]; 1 Bastion outlier removed).

We found the same pattern of effects for change in rs-fMRI connectivity between DMPFC and right amygdala: improved empathic accuracy from pre-intervention to post-intervention was associated with inreased rs-fMRI connectivity in Crystals participants compared to Bastion (*t*(59) = 2.42, *p* = 0.02, *b* = 0.96, CI [0.17, 1.70]; 1 Crystals and 1 Bastion outlier removed; Fig. 3b), and the relationship was significantly positive within Crystals (*t*(29) = 2.48, *p* = 0.02, *b* = 0.79, CI [0.14, 1.40]). There was no statistically significant relationship between changes in empathic accuracy and DMPFC—amygdala rs-fMRI for Bastion participants (*t*(31) = −0.70, *p* = 0.95, *b* = −0.02, CI [−0.52, 0.49]; 1 outlier removed). There was no group difference in change in DMPFC – amygdala rs-fMRI (*t*(62) = 0.10, *p* = 0.92, *b* = 0.01, CI [−0.18, 0.20]; 1 Crystals outlier removed). There were no baseline group differences in DMPFC—amygdala rs-fMRI connectivity (*t*(62) = 1.42, *p* = 0.16, *b* = 0.10, CI [−0.04, 0.25]; 1 Crystals outlier removed) or its relationship with empathic accuracy (*t*(59) = 0.19, *p* = 0.85, *b* = 0.07, CI [−0.69, 0.84]; 1 Crystals and 1 bastion outlier removed).

We also conducted a whole-brain voxel-wise analysis and there were no regions with significantly different change in connectivity between groups for the VMPFC or DMPFC seed, nor for group differences in change in VMPFC or DMPFC connectivity in relation to change in empathic accuracy.

### Group differences in empathic accuracy task-related activation

In order to test for group differences in empathic accuracy-related blood oxygenation level-dependent (BOLD) activation we modeled the linear relationship between the trial-wise activation estimates and the empathic accuracy score for each trial (see Methods for additional details on the empathic accuracy paradigm and behavioral measure). Beta estimates were extracted from each of three independently defined functional ROIs (VMPFC, DMPFC, and RTPJ)—the same ROIs as were used as seeds in the rs-fMRI analysis. We tested for group differences in change scores using a two-sample *t*-test. The 3 ROIs were chosen based on regions in which activation related to empathic accuracy in an independent sample of participants using the exact same task (drawn from baseline data from participants randomized to one of the other 2 conditions not analyzed in this study), and which have also been reported to be active with greater empathic accuracy in the literature.^16,22^

There were no group differences in change in empathic accuracy-related brain activation for any of the 3 ROIs: VMPFC (*t*(69) = 0.76, *p* = 0.45, *b* = 0.23, CI [−0.38, 0.84]; 1 Crystals and 1 Bastion outlier removed), DMPFC (*t*(70) = −1.71, *p* = 0.09, *b* = −0.33, CI [−0.72, 0.06]; 1 Crystals outlier removed) and RTPJ (*t*(70) = 0.13, *p* = 0.90, *b* = 0.03, CI [−0.42, 0.47]; 1 Crystals outlier removed). We also conducted a whole-brain voxel-wise analysis and there were no regions with a significant group difference in change in empathic accuracy-related brain activation.

### Effects of game-play on EA and related activation

We tested whether individual differences in Crystals’ game-play were associated with changes in brain activation in relation to empathic accuracy using the difference in post-intervention and pre-intervention BOLD activation from empathy trials that were modulated by trial-wise empathic accuracy scores and contrasted with implicit baseline (fixation). We extracted empathic accuracy-modulated BOLD response from each of the 3 ROIs (as described in the ROI analysis above) and regressed the difference scores (time 2 − time 1) onto the normalized average of the number of attempts at emotion recognition and emotion intensity ratings (that were collected from Crystals gameplay). The average number of first attempts at emotion recognition was 53 (range 27–74) and the average number of emotion intensity ratings was 29 (range 8–60). More first attempts at emotion recognition were associated with more completed intensity ratings (*t*(29) = 11.67, *p* < 0.001, *b* = 1.14, CI [0.94, 1.34], r = 0.91; 3 outliers removed). These measures provide a proxy for participants’ engagement with the training aspects of Crystals, and since they are so strongly related we combined them into a single game engagement score by computing the mean of the normalized variables, which included mean-centering and scaling by the standard deviation. Since these values are the number of first attempts, they reflect the number of times participants engaged with the training aspects of the game, rather than repeated tries due to inaccuracy. Although we planned to examine the relationship between changes in EA-related activation and accuracy on the empathy training game mechanics, the accuracy measures for each of these variables lacked sufficient variability across participants with which to make meaningful comparisons due to ceiling effects. The average accuracy for emotion recognition was 90% (range 73–100%) and for the emotion intensity ratings the average accuracy was 97% (range 83–100%).

**Fig. 4:**
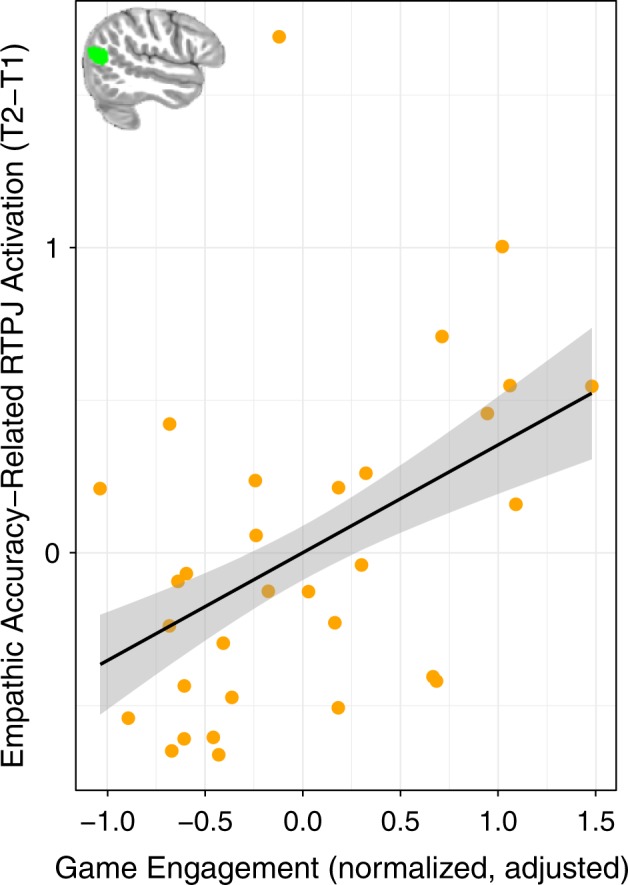
Empathic accuracy-related BOLD activation and engagement with the Crystals training video game. Participants who engaged more with the training aspects of Crystals had increased empathic accuracy-related RTPJ activation following game-play. Game engagement is the mean of the normalized values for number of first tries at emotion recognition and intensity calibration. Raw data points were adjusted for total time playing the game. Envelopes represent 1 standard error from the point estimates. The independently-defined ROI is inset in green

Engagement with the training aspects of Crystals was associated with significantly higher increases in empathic accuracy-related RTPJ activation (*t*(30) = 2.92, *p* = 0.01, *b* = 0.47, CI [0.14, 0.79]; 1 outlier removed; Fig. 4) while controlling for total time playing the game. This relationship remains consistent without the covariate for total playtime, however it is important that our results show an effect of the training mechanics above and beyond total time spent playing the game. Moreover, the total time playing Crystals was not associated with change in empathic accuracy-related RTPJ activation (*t*(31) = −0.21, *p* = 0.84, *b* < −0.01, CI [−0.01, 0.004]; 1 outlier removed). Aspects of the game that were either un-related to empathy training or did not directly target empathy-related processes did not predict change in empathic accuracy-related RTPJ activation (while controlling for total time playing), such as: the number of times using a taser (*t*(29) = 0.90, *p* = 0.38, *b* = 0.01, CI [−0.02, 0.04]; 1 outlier removed) and the number of quests completed—a generically pro-social aspect of the game that did not specifically target empathy-related processes (*t*(28) = 0.79, *p* = 0.44, *b* = 0.01, CI [−0.02, 0.04]; 2 outliers removed). Participants played Crystals for an average of 217 min (range 98–334 min) over the course of the training period. We were unable to examine changes related to gameplay in the control group, as we could not access gameplay data from Bastion. Change in empathic accuracy-related activation from the other 2 ROIs was not related to engagement with the game training tools (while controlling for total play time): VMPFC (*t*(29) = 1.35, *p* = 0.19, *b* = 0.32, CI [−0.17, 0.81]; 1 outlier removed) and DMPFC (*t*(28) = −1.14, *p* = 0.26, *b* = −0.14, CI = [-0.38, 0.11]; 2 outliers removed). There was no relationship between game engagement and change in empathic accuracy controlling for total playtime (*t*(30) = 1.02, *p* = 0.32, *b* = 0.06, CI [−0.06, 0.18]; 1 outlier removed).

We also conducted a whole-brain, voxel-wise analysis of change in empathic accuracy-related BOLD activation and there were no regions that were significantly associated with game engagement.

## Discussion

This study created and tested behavioral and brain effects of a video game intervention with mechanics to train empathy in an immersive virtual world. Another unique aspect about the empathy training video game developed for this study was the collection of in-game telemetry data that provided measures of engagement with and performance on the empathy training game mechanics. We were able to determine which aspects of Crystals related to individual differences in improvements following gameplay using these metrics. In addition to the empathy training game, Crystals, we also employed the commercially available game Bastion as an active control in this randomized controlled trial. This study design provided a rigorous method to test for group differences in empathic accuracy, the related brain activation and rs-fMRI connectivity. While there was no group difference in change in the behavioral measure, participants had sufficient variability in change in empathic accuracy scores for us to investigate brain activation related to increased empathic accuracy. We found that participants who engaged more with the empathy training game mechanics in Crystals (emotion recognition and gauging emotion intensity) had increased empathic accuracy on trials in which they also increased in RTPJ activation (from pre-intervention to post-intervention).

The ROIs (VMPFC, DMPFC and RTPJ) were defined using a functional localizer (the empathic accuracy task) in an independent sample of adolescents, and were chosen because activation in these regions has been associated with greater empathic accuracy at baseline in our full sample of adolescents^22^ and separately in adults.^16^ Activation in these regions appears to underlie cognitive aspects of empathic responding, as these regions have been strongly implicated in perspective taking, and in inferring goals and end-states as part of a broader brain network.^10,13,35,37,38,39^ Thus, playing Crystals may train participants to more effectively recruit a region involved in perspective taking (RTPJ) to make empathically accurate responses. One mechanism by which this change may have occurred is through rehearsal of taking the avatars’ perspectives through consideration of the narrative context in the game, which could facilitate accuracy on subsequent recognition and gauging intensity of the avatars’ emotions.

We also found that playing Crystals led to increased rs-fMRI connectivity within this same network—between DMPFC and PCC—compared to Bastion. The change in functional connectivity was specific to this network, as whole-brain analysis did not reveal any additional regions where the groups differed in connectivity with any of the seed regions (DMPFC, VMPFC, or RTPJ). Given that Crystals included training to recognize and attend to the emotions and thoughts of others, and the well-documented role of this network in mentalizing/perspective taking^18,35,40^ in addition to being immediately relevant for empathic accuracy in this study, we interpret the strengthened connectivity to reflect training and improvement in perspective taking ability following Crystals gameplay. Rehearsal of perspective taking during the game, paired with positive reinforcement of subsequent correct responses, may have facilitated learning and strengthened the brain network that supports perspective taking.

In addition to examining rs-fMRI connectivity changes within the network underlying empathic accuracy in adolescents, we examined changes in amygdala—MPFC rs-fMRI connectivity, which has been implicated in emotion regulation.^41^ This analysis was motivated by the expectation that strengthened connectivity in networks underlying emotion regulation would be associated with improvements in empathic accuracy, as individuals with better self-regulatory abilities more often experience empathic care rather than distress.^25^ As we previously reported that adolescents in this study had lower empathic accuracy with activation in regions underlying a shared emotional experience (at baseline),^22^ and as such experience sharing can lead to empathic distress through self-focus,^23–25^ we expected that improvements in emotion regulation would be particularly beneficial for adolescents’ empathic accuracy. Indeed, we found that Crystals participants who had strengthened VMPFC and DMPFC rs-fMRI connectivity with amygdala after video game training also had improvements in empathic accuracy compared to Bastion participants. Although the current study lacked direct measures of participants’ emotion regulation abilities, the strong evidence for connectivity in this network as underlying implicit emotion regulation,^41^ together within the context of its relation to behaviorally-relevant, hypothesized improvements in empathic accuracy lends credibility to this interpretation.

While playing the empathy training game Crystals did not lead to a change in the behavioral measure of empathic accuracy across participants, this may be due to the fact that most participants found the game to be relatively easy. However, those participants who showed improvements in empathic accuracy also showed increased self-reported empathic concern in addition to the multiple measures of changes to relevant brain networks described above. One limitation of the current study was the relatively low level of difficulty of Crystals, as evidenced by ceiling effects in accuracy on all of the empathy training game mechanics. The game difficulty could be improved by using subtler emotion expressions and a greater variety of emotion categories and contexts. The game’s difficulty could also be calibrated based on participants’ performance, which may create space for improvement within the game and on the empathic accuracy task. Conversely, Crystals may be more beneficial for those with difficulties at reading emotional expressions and empathizing, such as individuals with autism.^42,43^ Future research could explore whether Crystals’ empathy training is effective for improving empathic abilities for individuals on the autism spectrum.

While this research is in the early stages, these initial results provide evidence that video games could be utilized to improve empathy-related brain function and connectivity in adolescents through skillful use of game mechanics that tap into processes underlying empathy, specifically perspective-taking and emotion regulation. Additional research with clinical populations and/or participant-adaptive game difficulty is needed to elucidate whether such training could also lead to improvements in empathic behavior. This platform has the potential to be widely accessed and readily consumed by adolescents who are eager to use the newest electronic entertainment, and parents who would like positive alternatives to the commonly available games. The benefits of training empathy include the possibility to improve social interactions during a time of life when social support could be critical to overcoming hardship and improving wellness.

## Methods

### Participants

We advertised in print, broadcast and online media to recruit 192 healthy adolescents (average age 12.8 years ± 8.8 months, age range 11–15 years, 69 female) from the Madison, WI community between June 2013 and April 2014. Participants had to be enrolled in the 7th or 8th grade, fluent English speakers, safe for MRI scanning, not using psychotropic mediations, and with no current or previous diagnosis of a mental illness. Thirty-three participants’ data were unusable due to technical issues (21), inability to see or hear the stimuli (8), or not completing the task (4). Participants were randomly assigned to play one of four games—two games for the current study and a different set of two games for a separate study on attentional training—after completing baseline data collection using block randomization in sets of eight. The randomization procedure and intervention assignment were conducted by the project manager and a research assistant in a password-locked spreadsheet that restricted data collectors and other study personnel from access and utilized a random number generator to create the randomization sequence with a different number to seed the generator for each block. A different full-time research assistant conducted participant enrollment. The sample size was determined using a power analysis with the fmripower tool^44^ based on effect size estimates from the original empathic accuracy fMRI study^16^ from which the task used in the current study was designed. Two of the games were intervention games designed for this study, and the other two games were commercially available and served as control conditions with similar play styles to one of the two intervention games. Approximately half of the participants (*N* = 82, average age 12.9 years ± 9.0 months, 30 female) played the empathy training game (described below) or its commercial control game, Bastion. Participants were instructed to play the game on their own (i.e., rather than collaboratively with peers). The remaining 77 participants played one of two games not hypothesized to affect empathic processes. Data from those subjects were used to define independent regions of interest for tests of group differences in functional MRI data described below. At post-intervention (following 2 weeks of daily game-play) 8 additional participants’ data was unusable (2 left the study, 3 due to technical difficulties, and 3 were non-compliant with the intervention). This left 34 participants who played the empathy training game Crystals (average age 12.9 years ± 8.8 months, 14 female) and 40 participants who played the control game, Bastion (average age 12.8 years ± 8.6 months, 13 female). An additional 3 Crystals and 7 Bastion participants were excluded from rs-fMRI analysis due to technical difficulties and excessive motion that made either one of the two scans unusable. Analysis of baseline data across the full sample of participants was previously reported.^22^ Experimenters were only completely blind to group assignment during baseline data collection due to practical limitations of administering the intervention. UW-Madison’s Health Sciences Institutional Review Board approved the protocol, and all participants provided informed assent and were given monetary compensation for their participation. Legal guardians provided written informed consent. All data was collected at the Waisman Center at UW–Madison, Madison, WI between July 2013 and November 2014.

### Questionnaires

Participants completed a battery of questionnaires as part of a larger study, which included measures of cognitive control and response inhibition to assess the impact of a separate attention training videogame. The Interpersonal Reactivity Index (IRI^32^) contains 28 items and 4 sub-scales with 7 items each, one of which measures a construct related to empathy that is relevant to the current report: empathic concern. The test–retest reliability of the IRI is good, with intra-class correlations ranging from 0.61 to 0.81 over 60–75 days.^45^

### Empathic accuracy task

The empathic accuracy task used in the current study was previously described in Kral et al. (Supplementary Figure 3).^22^ Stimuli in the task consisted of videos of targets (aged 18–21 years) describing emotional events from their adolescence, such as the death of a grandparent or winning a sports competition. Targets watched their own videos and made ratings of their emotions as displayed in the videos on a 1–9 Likert scale, with 1 labeled “Very Negative”, and 9 labeled “Very Positive”.

Participants in the current study (“perceivers”) completed the empathic accuracy task twice; first during a baseline data collection session and again following 2 weeks of daily game-play with either Crystals or Bastion (the active control). The task occurred in 3 fMRI scan runs, each lasting approximately 5 min. A cue word was presented for 3 s, followed by a fixation cross for 2 s and then a video, which ranged from 28 to 144 s (mean = 90 s). There were two sets of videos so that participants saw a separate, unique set of videos at baseline and post-intervention, and the order of presentation across scans was counter-balanced across participants. The same targets appeared in both sets of videos describing distinct events. A cue instructed participants how to rate the videos, corresponding to three different conditions. If the word “OTHER” appeared participants were instructed to rate the emotion of the target in the video from negative to positive, exactly as the targets had rated themselves. If the word “SELF” appeared participants were instructed to rate their own emotions from negative to positive using the same scale. In the control condition the word “GAZE” appeared and participants were instructed to rate the direction of the target’s eye-gaze from left to right using a 1–9 Likert scale with 1 labeled “Left” and 9 labeled “Right”. The current report focuses on responses in the “OTHER” condition, which correspond to empathic accuracy trials. For each scan the order of trial conditions was pseudo-randomized such that perceivers saw a different order of 6 trials in each of the 3 blocks of the task, and targets were half male and half female. There were 18 trials, 6 per condition, and each had a unique video stimulus. Thus, participants rated 36 videos over the course of the study, and 12 total videos for the empathic accuracy analysis. Prior to each scan participants completed three practice trials with a set of videos not used in the fMRI task. Empathic accuracy was determined by calculating the correlation between the time course of ratings for the target and the perceiver for each video and r- to Z-transforming with Fisher’s method. One trial was excluded from all participants due to large disagreement between the target’s rating and the majority of perceiver ratings, which resulted in extremely low average empathic accuracy scores across perceivers (*r* < 0.10).

### Video game training intervention

In the empathy training game, Crystals, players control a robot that has crash-landed on an alien planet and must learn how to understand, communicate with, and ultimately collaborate with the inhabitants to find components of the damaged spaceship in order to return home (Fig. 1a). Crystals includes game mechanics to train recognition of the six basic emotions (anger, fear, happiness, surprise, disgust, and sadness; Fig. 1b), to gauge varying levels of emotion intensity (Fig. 1c), to respond empathically (Fig. 1d) and practice helping behaviors in the virtual world (Fig. 1f). The commercial game Bastion (version 1.2) was selected as an active control since it has a similar story-based environment, immersive graphics and third-person perspective. In-game performance (i.e., telemetry data) was recorded for Crystals using Assessment Data Aggregator for Game Environments (ADAGE).^46,47^

### Image acquisition

Images were acquired on a GE X750-3.0 Tesla MRI scanner (Waukesha, WI) with a 32-channel head coil (Nova Medical Inc., Wilmington, MA). Anatomical scans consisted of a whole head 1.0 mm isotropic T1-weighted image acquired using an in-house MPnRAGE sequence.^48^ Three functional scan runs were acquired for the empathic accuracy paradigm using a gradient echo EPI sequence (64 × 64 in-plane resolution, 220 mm FOV, TR/TE/Flip = 2000ms/20 ms/60°, 36 × 4.5 mm), and one resting state functional MRI run was acquired with 264 time points using the same EPI sequence.

### Image analysis

See supplementary methods for pre-preprocessing information.

Three ROIs (VMPFC, DMPFC, RTPJ) that were previously implicated in empathic accuracy in adults^16^ were functionally defined based on regions with greater empathic accuracy-related activation in an independent sample of same-age participants at baseline (pre-intervention) that were randomly assigned to play a game not included in the current analysis. ROIs were transformed from Montreal Neurological Institute (MNI) space to the group average space via a transformation matrix created using ANTS for analysis,^49^ however all results are displayed on the MNI template brain. A mask combining all regions with activation related to empathic accuracy in the independent sample was also created for use in a voxel-wise rs-fMRI analysis to examine changes in connectivity within this network (see Fig. 2). For rs-fMRI connectivity analysis with each of the seed regions (VMPFC, DMPFC, and RTPJ) the seed was subtracted from the mask in which the analysis was conducted (for depiction of the ROI masks see Fig. 3 for VMPFC and DMPFC, Fig. 4 for RTPJ). The right amygdala ROI was defined anatomically based on the Harvard-Oxford atlas (distributed with FSL^50^) at 50% threshold (see Fig. 3).

### Statistical analysis

Resting state fMRI connectivity was assessed based on the Fisher Z normalized correlation between each of the seeds and each of the target ROIs (or for each voxel in the voxel-wise analysis). In order to test for intervention effects, we computed difference maps for the post-intervention FZT-map subtracting the pre-intervention FZT-map. Average correlation values were extracted from each of the ROIs for analysis with the lm package in R Statistics,^51^ and voxel-wise analysis was also conducted whole-brain and within the masks specified above. Voxel-wise analyses were thresholded at *p* < 0.05 controlling for family-wise error using threshold-free cluster enhancement with FSL’s Randomize.^34^

This study is registered as a clinical trial with ClinicalTrials.gov (number NCT01886911) and the trial ended upon planned completion of data collection.

### Code availability

Code for statistical analysis can be found at: https://osf.io/kveyp/. Enquiries about code access can be made to Tammi Kral, M.S. (kral@wisc.edu).

### Data availability

Enquiries about data access can be made to Dr. Richard Davidson (rjdavids@wisc.edu).

## Electronic supplementary material

Supplementary Methods

Supplementary Figures

## Electronic supplementary material

**Supplementary information** accompanies the paper on the *npj Science of Learning* website (10.1038/s41539-018-0029-6).
