# Supplementary material for: Neural correlates of video game empathy training in adolescents: a randomized trial
Source: NPJ Sci Learn. 2018 Aug 7;3:13. doi: 10.1038/s41539-018-0029-6 (PMC6220300; doi:10.1038/s41539-018-0029-6)
Supplement: Supplementary file 1 — Supplementary Methods [file 41539_2018_29_MOESM1_ESM.docx]

**Supplementary methods.**

**Image pre-processing.**

*Empathic Accuracy task:*

FMRI data processing was carried out using FEAT (FMRI Expert Analysis Tool) Version 6.00, part of FSL (FMRIB's Software Library, www.fmrib.ox.ac.uk/fsl). The following pre-statistics processing was applied; motion correction using MCFLIRT;^52^ non-brain removal using BET;^53^ spatial smoothing using a Gaussian kernel of FWHM 5mm; grand-mean intensity normalization of the entire 4D dataset by a single multiplicative factor; highpass temporal filtering (Gaussian-weighted least-squares straight line fitting, with sigma=50.0s). The functional data from individual subjects was analyzed using a General Linear Model (GLM) in 3 levels, where the first level modeled the data within-run using FILM prewhitening,^54^ the second level combined data within-subject separately for each trial using a fixed effects modeling approach and the third level modeled data across subjects using the Flame1 mixed effects model estimation. In the first level each video was modeled as a separate block regressor, with length equal to the video duration, and each cue period was modeled as a block for the 3 s duration while the fixation period was un-modeled and contributed to the baseline. All regressors were convolved with a double gamma hemodynamic response. Additional regressors of no interest were included to model 24 total motion-related parameters (the standard plus extended parameters, which include the squares, derivatives and squares of derivatives), as well as trials during which participants did not make ratings, and the single trial that was excluded across all participants (as described above). To further address motion, high motion time points were modeled out of the data with an individual regressor that consisted of all 0's and a single 1 for the TR of interest. High motion time points were identified as having a framewise displacement (FD) measure larger than 0.9 mm.^55^ Runs with more than 25% (77 TRs) of the data censored were omitted from analysis, leading to a total of 6 omitted runs. On average 7 TRs were censored (interquartile range 0, 6).

Contrasts were created at the second level that estimated the linear relationship between the block-based BOLD activation and within-block empathic accuracy in a within-subject, fixed effects analysis. Only the 6 estimates for the "OTHER" trial blocks in which participants rated the target’s emotions provided a measure of empathic accuracy and were considered in higher-level analyses. Functional data were aligned to a group average anatomical space, which was created using Advanced Normalization Tools (ANTs).^56^ Image registration of the BOLD data to the study-specific group template was carried out using FSL in a two stage process where the Boundary Based Registration (BBR) approach,^57^ was used to register the subject's time series data to their anatomical template and a 12DOF affine transformation was used to register the subject's anatomical to the group template using FLIRT.^52^ The group analysis then estimated the average of this linear slope across subjects in a mixed effects Flame 1 analysis. The number of ratings made per minute for each trial was an additional regressor of no interest to control for differences in the amount of ratings.

*Resting state fMRI*

Data processing and analyses were performed using AFNI^58^ analysis package, unless otherwise indicated. Reconstructed echo-planar image volumes were first corrected for motion using rigid-body realignment (3dvolreg) and corrected for slice-timing differences (3Tshift). The first 3 images (6 s) were ignored to allow magnetization to reach equilibrium. Data were then corrected for B0-field distortions using customized in-house software that calls the FMRIB Software Library, FSL^59^ functions PRELUDE and FUGUE. This data was then aligned to the T1-weighted structural using an affine transformation and a local Pearson correlation cost function.^60^ T1-weighted structural data was aligned to the MNI template using nonlinear warping, ANTS.^56^ This warp was then applied to the preprocessed fMRI data, and resampled to 2 mm isotropic resolution. Automated segmentation (FSL’s FAST) of the T1-weighted structural image was used to define masks of the WM and CSF.^54,59,61^

The two signal intensity time-courses resulting from averaging the fMRI data within the eroded WM and CSF masks as well as their first derivatives (computed by backwards difference) were taken as signals of no-interest (i.e., spurious fluctuations unlikely to be of neuronal origin) and removed from the functional data along with the six rigid-body motion registration parameters.^62,63^ Time points where the sum-squared difference (ssd) of consecutive points of the 6 motion realignment parameters exceeded 0.25 mm were censored and ignored in this nuisance regression. Participants with greater than 25% of data censored at either scan (pre- or post-intervention) were excluded from analyses due to insufficient total scan data. The functional images were temporally band-pass filtered between 0.01 Hz and 0.1 Hz, and spatially smoothed with a 3-dimensional Gaussian kernel (FWHM = 6 mm).

Functional connectivity was computed using a seed-region based approach.^64^ For each seed region, the preprocessed fMRI data was averaged over the seed region of interest, and then regressed against all voxels in the brain. Again, time points with excessive motion (ssd > 0.25 mm) were censored and ignored in this regression analysis.
