# Supplementary material for: Neural correlates of video game empathy training in adolescents: a randomized trial
Source: NPJ Sci Learn. 2018 Aug 7;3:13. doi: 10.1038/s41539-018-0029-6 (PMC6220300; doi:10.1038/s41539-018-0029-6)
Supplement: Supplementary file 2 — Supplementary Figures [file 41539_2018_29_MOESM2_ESM.pdf]

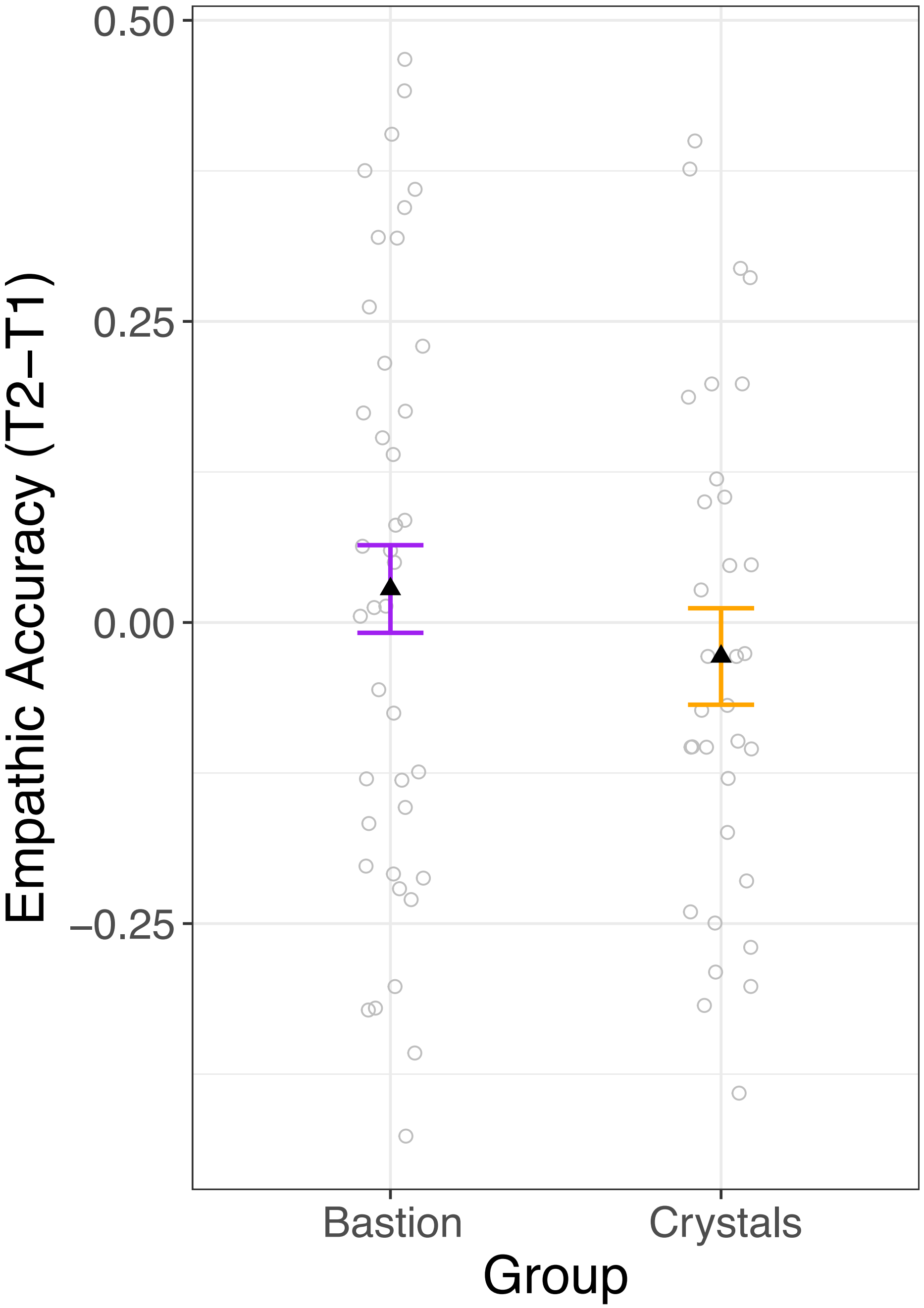

Supplementary Figure 1. Change in empathic accuracy. There were no changes or group differences in change in empathic accuracy from pre- to post-intervention. Error bars represent 1 standard error from the point estimates of the means, and raw data points are overlaid in gray.

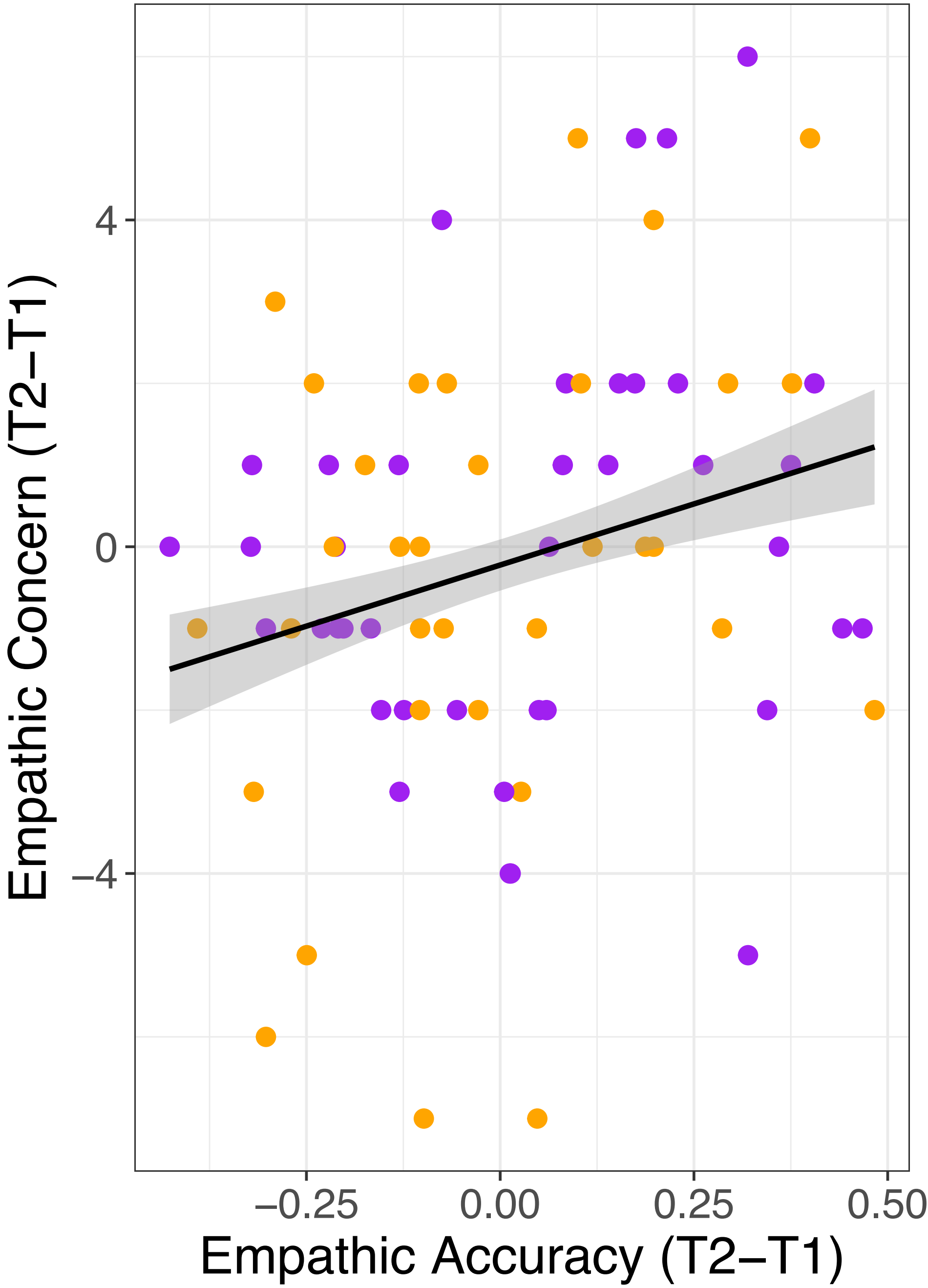

Supplementary Figure 2. Relation between changes in self-report and behavioral empathy measures. Increased empathic accuracy was associated with increased self-reported empathic concern from pre- to post-intervention. Envelopes represent 1 standard error from the point estimates and raw data points are overlaid in purple for Bastion and gold for Crystals.

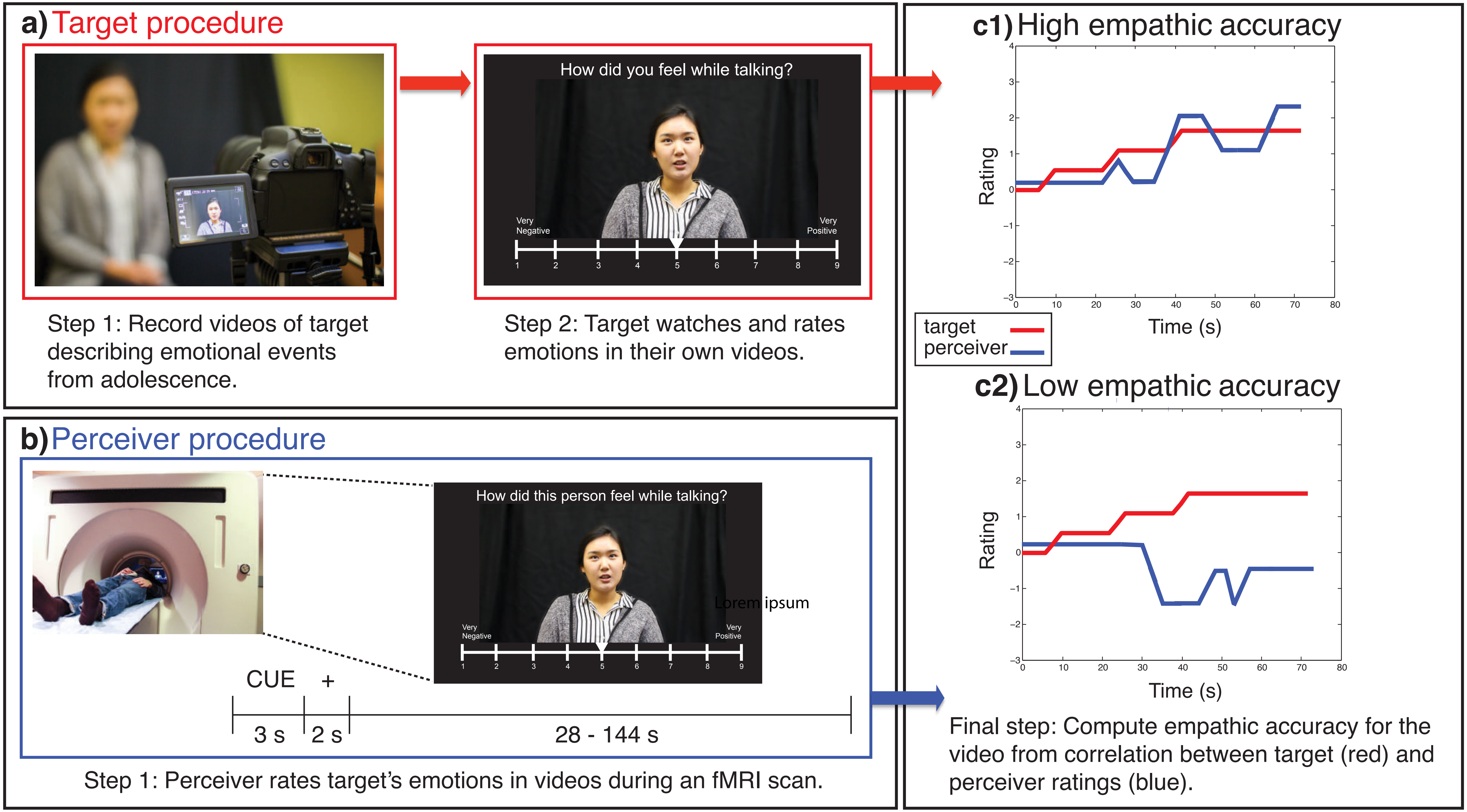

Supplementary Figure 3. Empathic accuracy task. Targets were video-recorded describing emotional autobiographical events, and subsequently rated their emotions as displayed in the videos from negative to positive (a). Participants in the current study, "perceivers", watched the same videos of targets during a functional MRI scan, and rated the targets' emotions from negative to positive (using the same scale; b). Ratings were collected continuously during the videos. Empathic accuracy was computed by taking the correlation between the timecourses of target and perceiver ratings of the targets' emotions for each video. High empathic accuracy is indicated by a strong correlation and reflects higher agreement between target and perceiver, while low empathic accuracy is indicated by a weak correlation and reflects little agreement between target and perceiver (c). This figure is adapted with permission from Oxford University Press, and was originally published in *SCAN* (Kral et al., 2017).
